# Plant viruses as probes to engineer tolerance to abiotic stress in crops

**DOI:** 10.1007/s44154-022-00043-4

**Published:** 2022-04-05

**Authors:** Emmanuel Aguilar, Rosa Lozano-Duran

**Affiliations:** 1 Shanghai Center for Plant Stress Biology, Chinese Academy of Sciences, Shanghai, 201602 China; 2 Center for Excellence in Molecular Plant Science, Chinese Academy of Sciences, Beijing, China; 3 Departamento de Biología Celular, Genética y Fisiología, Instituto de Hortofruticultura Subtropical y Mediterránea “La Mayora”, Universidad de Málaga-Consejo Superior de Investigaciones Científicas (IHSM-UMA-CSIC), Universidad de Málaga, Campus Teatinos, 29071 Málaga, Spain; 4 Department of Plant Biochemistry, Centre for Plant Molecular Biology (ZMBP), Eberhard Karls University, D-72076 Tübingen, Germany

## Main text

Plants, as sessile organisms, have to deploy a vast array of mechanisms to cope with the always-changing environmental conditions that surround them. The increasing accumulation of greenhouse gases in the atmosphere in the last century is responsible for a global warming of which the effects are predicted to be magnified in the near future. As a consequence of more unstable climatological conditions in a warmer world, the Intergovernmental Panel on Climate Change (IPCC) predicts a higher frequency and intensity of extreme phenomena, such as long periods of drought and high temperatures, affecting vast regions of the planet (IPCC, 2021). In this context, the study of the strategies plants deploy to face these environmental challenges, and the use of this knowledge for the generation of stress tolerant plants, will be paramount to ensure food security for the growing world population. Particularly urgent is the identification of strategies to promote resilience to climatic-related stresses such as high temperature and drought.

Although the mechanisms underlying abiotic stress responses in plants have been extensively studied in the past few decades, our understanding of these complex physiological processes and of the diverse strategies conferring tolerance to these cues is only partial. Most studies have been conducted with the aim to elucidate the general responses plants activate under individual, specific stresses, by assuming the same mechanisms will still operate or trends will be similar in more complex situations. Although key to generate a basic picture of the mechanisms involved in plant tolerance to stress, this approach has proven to be too simplistic. The fact that plants have to face not only the aforementioned abiotic stress factors, most frequently in combination, but also the challenge of multiple biotic factors concomitantly, makes this picture necessarily more complex, and the resulting responses generally unpredictable by analyzing each factor individually (Prasch and Sonnewald, 2013). Among the biotic factors affecting plants, viruses constitute one of the major threats to crop production worldwide; therefore, analyses considering plant-virus interactions under stress factors associated with climate change are of capital importance.

**Fig. 1:**
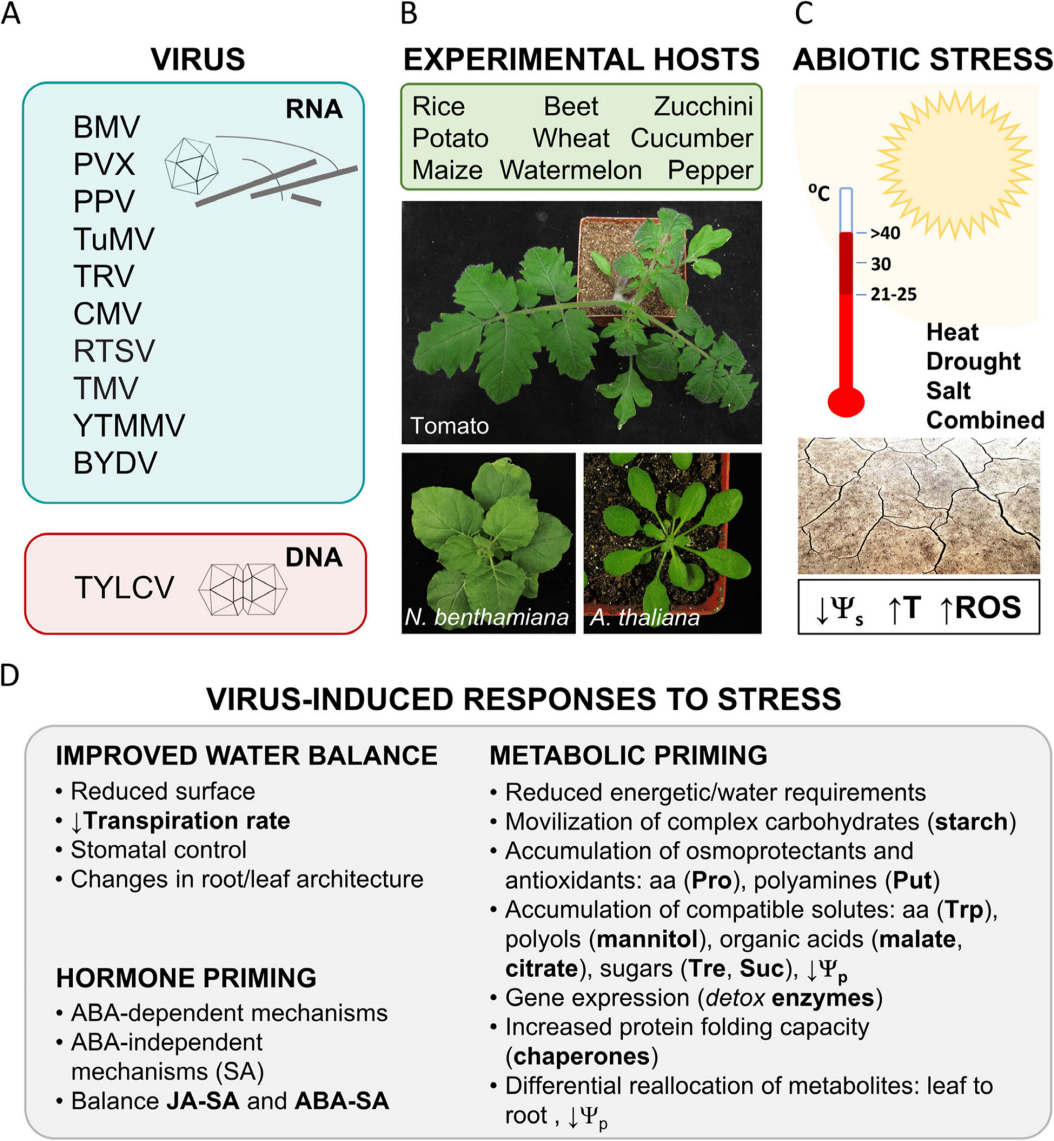
Virus-induced tolerance to abiotic stress. **A**. Induction of tolerance to abiotic stress has been described in plant-virus interactions involving different genera of RNA (+) viruses and, at least, one ssDNA virus. **B**. Viral-induced tolerance has been analyzed in the usual experimental hosts *Nicotiana benthamina*, *Arabidopsis thaliana*, and tomato, and in many crop species. **C**. The occurrence of different abiotic stresses (heat, drought, salt), considered individually or combined, result in a reduction of the soil water potential (Ψ_s_), temperatures exceeding the optimal limits for the vegetative and reproductive growth (↑T), and higher oxidative potential (ROS). **D**. Under these challenging environmental conditions, virus-infected plants exhibit and array of virus-induced responses which mainly result in i) a reduction of the plant water potential (Ψ_p_) below Ψ_s_, allowing for an easier water uptake, and in ii) a better capacity to manage with the oxidative stress, among others; bold letters denote beneficial responses described for most of the plant-virus interactions analyzed to date. SA, salicylic acid; JA, Jasmonic acid; ABA, Abscisic acid; Put, Putrescine; Pro, Proline; aa, amino acids; Trp, Tryptophan; Tre, Trehalose; Suc, Sucrose. BMV, brome mosaic virus (*Bromovirus*); PVX, potato virus X (*Potexvirus*); PPV, plum pox virus (*Potyvirus*); TuMV, turnip mosaic virus (*Potyvirus*); TRV, tobacco rattle virus (*Tobravirus*); CMV, cucumber mosaic virus (*Cucumovirus*); RTSV, rice tungro spherical virus (*Waikavirus*); TMV, tobacco mosaic virus (*Tobamovirus*); YTMMV, yellow tailflower mild mottle virus (*Tobamovirus*); BYDV, barley yellow dwarf virus (*Luteovirus*); TYLCV, tomato yellow leaf curl virus (*Begomovirus*); *N. benthamiana* (*Nicotiana benthamiana*); *A. thaliana* (*Arabidopsis thaliana*)

Traditionally, plant viruses have been considered obligate intracellular ‘parasites’ but, in the last few years, thanks to the advancement of disciplines such as Viral Ecology and Metagenomics, a new vision is emerging, in which viruses are prevalent in the absence of disease. This new vision considers viruses more than pathogenic elements: viruses are biological entities able to establish a diverse array of interactions with their host, ranging from the canonical parasitism to a mutualistic beneficial symbiosis, depending on the environmental situation in which this interaction takes place (Roossinck 2011, 2015). Many different viruses, belonging to different genera, and encompassing both DNA and RNA viruses, have been shown to establish a beneficial relationship with their host when the environmental conditions are restrictive (González et al., 2020). Xu and coworkers (2008) showed how several RNA viruses, such as cucumber mosaic virus (CMV), tobacco mosaic virus (TMV), or bromo mosaic virus (BMV), infecting crop species such as beet, tobacco, or rice, were able to significantly delay the appearance of drought-associated symptoms in their hosts following water withdrawal. This effect has also been described in wood crops, such as grapevine infected with grapevine fanleaf virus (GFLV) (Jez-Krebelj et al., 2022). Infection by a DNA virus (tomato yellow leaf curl virus, TYLCV) has also been found to confer both thermotolerance and drought resistance to tomato plants (Anfoka et al., 2016; Corrales-Gutierrez 2020; Mishra et al., 2021; Shteinberg et al., 2021) (Fig. 1). Examples of the opposite situation, in which the viral infection causes a reduction in the tolerance to drought, although scarce, have also been reported; such is the case of the interactions between *Arabidopsis thaliana* (hereafter referred to as Arabidopsis) and cauliflower mosaic virus (CaMV, DNA virus) (Bergès et al., 2018) or turnip mosaic virus (TuMV, RNA virus) (Manacorda et al., 2021).

The evolutionary aspects of the trade-offs established between viral infection and host performance, shaped by the environmental conditions, have recently been analyzed by González and coworkers (2021). In this study, Arabidopsis was inoculated with TuMV, and subjected to serial passages to generate artificially-evolved viruses, in normal watering conditions or under drought stress; different natural accessions of Arabidopsis, where the virus exhibits different degrees of virulence, were also included in the analysis. These experiments showed how the drought-evolved TuMV was able to increase the tolerance of Arabidopsis to drought, while the TuMV evolved under standard conditions only did it marginally, with marked differences between accessions; these results exemplify how a virus can adapt under environmental pressures to shift its interaction with the plant from parasitism to mutualism, therefor benefiting both partners. Adding another layer of complexity, Aguilar and coworkers (2017) showed how most virulent infections were able to induce tolerance to drought more efficiently, but only infections with moderate levels of virulence, although inducing modest tolerance to drought, allowed the plant to complete its life cycle, generating viable offspring. In this sense, the general assumption that increased viral-induced tolerance may be always followed by increased biological efficacy (fitness, or seed production), also needs revisiting.

The presence of a viral infection entails a whole reprogramming of the plant transcriptome, methylome, and hormonal balance, leading to profound physiological and metabolic changes. From a theoretical perspective, viruses could trigger a vigorous activation of defence mechanisms the plant deploys to protect itself, which may be redundant and overlapping, leading to common responses under different stress situations. If this were the case, a plant could be prepared (primed) to cope with an abiotic stress after the establishment of the infection. This may be the explanation for most of the plant-virus interactions leading to increased tolerance to drought, which accounts for most of the interactions analyzed to date. Actually, the activation of common defence responses to different stress situations, such as the closure of stomata or the accumulation of salicylic acid, strongly suggests a contribution of such priming. On the other hand, a viral infection prior to the exposure to abiotic stress could result in decreased host tolerance as well, because of the accumulation of toxic byproducts generated during defence responses, or subsequent changes in hormone regulation, especially that of ABA: this assumption could be at the basis of the few reported plant-virus interactions leading to reduced drought tolerance (Manacorda et al., 2021).

One of the first responses triggered under water deprivation is stomatal closure, reducing water lost by evapotranspiration. Decreased transpiration rates under infection are commonly found in several plant-virus interactions, correlating with the increased tolerance to drought (Xu et al., 2008; Aguilar et al., 2017; Shteinberg et al., 2021). The control of the stomatal aperture may be exerted through non-canonical ABA-independent pathways, involving hormones such as SA, of which the accumulation also correlates with the induction of tolerance in some pathosystems (Xu et al., 2008, Aguilar et al., 2017). Transpiration can also be modulated in a different way: an increased occurrence of occluded vessels in infected plants, resulting in reduced transpiration rates, has recently been suggested as explanation for the induction of tolerance in GFLV-infected grapevines under moderate drought stress (Jez-Krebelj et al., 2022).

In Arabidopsis, on the other hand, the induction of strong drought tolerance in infected plants, in spite of higher transpiration rates than non-infected plants, points to the involvement of other mechanisms (Westwood et al., 2013, Aguilar et al., 2017). Drought stress is characterized by a reduction in the water availability in the soil, which implies a reduction in the soil water potential (Ψ_s_). Plants can respond to this change in two ways: by altering the root architecture in order to increase the surface available for water acquisition, or by decreasing the plant water potential (Ψ_p_), facilitating water uptake. Westwood and coworkers (2013) showed that transgenic expression of 2b, the viral protein responsible for the CMV-induced tolerance to drought in Arabidopsis, led to an increase in the number and size of lateral roots. On the other hand, a reduction in Ψ_p_ can be achieved by increasing the accumulation of compatible metabolites such as amino acids, sugars, and polyalcohols. In addition, these compounds can also act as osmoprotectants by stabilizing other macromolecules and cell structures during the desiccation process. Special relevance has the accumulation of proline (Pro) and polyamines such as putrescine (Put): Pro plays a multifunctional role, acting as osmoprotectant, buffering the pH and, together with Put, deactivating oxidative species. Viral infections also activate the accumulation of most of these compounds, which could explain the induction of tolerance to drought in plants with high transpiration rates (Fig. 1). Recently, Mishra and coworkers (2021) also described a TYLCV-induced reallocation of metabolites from shoot to roots that could be responsible for increasing tomato tolerance to drought. Something to note here, however, is the fact that, although common correlations can be found, plant metabolic profiles are dramatically different in different pathosystems (González et al., 2021).

The analyses conducted to date highlight the enormous potential of viruses as tools to promote and analyze tolerance mechanisms to abiotic stresses, including climatic perturbations, and delineate an alternative path to engineer more resilient plants. The ability of some viruses to induce tolerance seems to depend on specific proteins, such as 2b in CMV, C4 in TYLCV, or P25 in potato virus X (PVX), which raises the possibility of generating tolerant plants by the single expression of isolated transgenes (Westwood et al., 2013; Aguilar et al., 2017; Corrales-Gutierrez et al., 2020). These viral proteins are virulence factors whose transgenic expression sometimes imposes penalties to plant growth and development, limiting their practical applications. Gaining insight into the molecular mechanisms underlying the activities of these viral proteins might enable the generation of truncated forms or the isolation of new variants without negative effects, but still able to induce tolerance, hence offering an opportunity to avoid this trade-off. In more complex situations, where the enhancement of tolerance cannot be assigned to any particular viral protein, the isolation of attenuated or asymptomatic variants could also bring some benefits, at least in particular conditions (Encabo et al., 2020; Shteinberg et al., 2021), while avoiding the penalty that virulence imposes to plant fitness. Of note, in crops where the interest relies on the generation of vegetative tissues rather than in their reproductive performance, high virulence, achieved by selecting viral strains or by expressing key virulence factors, may generate higher levels of tolerance to drought. All things considered, and in view of the growing body of available data on the effects of plant-virus interactions, we propose that, in the current context of climate change, where ensuring food security for the ever-increasing world population is a top priority, plant viruses can offer an alternative route in the search for strategies to improve plant resilience to abiotic stress.

## Data Availability

Not applicable.

## Abbreviations

ABA: Abscisic acid
*A. thaliana*: *Arabidopsis thaliana*
BMV: Brome mosaic virus (*Bromovirus*)
CaMV: Cauliflower mosaic virus (*Caulimovirus*)
CMV: Cucumber mosaic virus (*Cucumovirus*)
DNA: Deoxyribonucleic acid
GFLV: Grapevine fanleaf virus (*Nepovirus*)
IPCC: Intergovernmental Panel on Climate Change
*N. benthamiana*: *Nicotiana benthamiana*
pH: Potential of Hydrogen ions
Pro: Proline
Put: Putrescine
PVX: Potato virus X (*Potexvirus*)
RNA: Ribonucleic acid
SA: Salicylic acid
TMV: Tobacco mosaic virus (*Tobamovirus*)
TuMV: Turnip mosaic virus (*Potyvirus*)
TYLCV: Tomato yellow leaf curl virus (*Begomovirus*)
Ψ_p_: Plant water potential
Ψ_s_: Soil water potential
